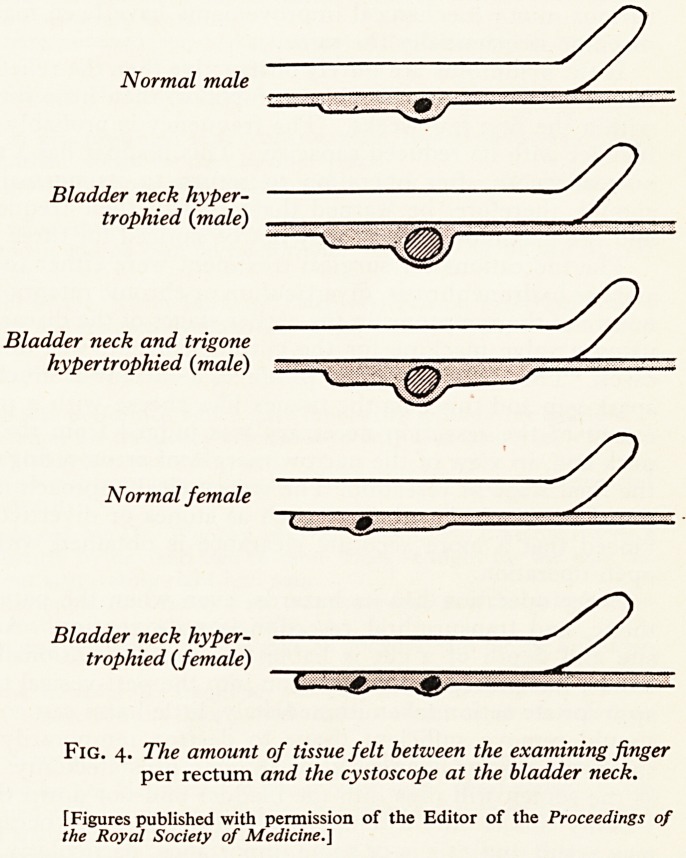# Congenital Bladder-Neck Obstruction
*Papers read to Bristol Medico-Chirurgical Society.


**Published:** 1956-01

**Authors:** Ashton Miller, J. P. Mitchell

**Affiliations:** United Bristol Hospitals; United Bristol Hospitals


### CONGENITAL BLADDER-NECK OBSTRUCTION*

BY

ASHTON MILLER, M.A., M.D., F.R.C.S. AND J. P. MITCHELL, M.S., F.R.C.S.
(*United Bristol Hospitals*)

### PART I

BY J. P. MITCHELL

Mr. Miller and I wish to thank you and the Society for your kindness in inviting us to speak to the Meeting tonight. We are very happy to have the opportunity of presenting this subject to you.

Obstruction at the outlet of the bladder may arise from a variety of easily recognized conditions such as hypertrophy or carcinoma of the prostate, but after excluding such conditions where the cause of the obstruction is obvious, there still remains a large number of cases of urinary dysfunction in whom some element of obstruction can be proved from the clinical and endoscopic findings. These cases have a normal prostate, are free of any anterior urethral stenosis and show no signs of neurological disease. By simple deduction the cause must be some intrinsic disorder of the complex sphincter mechanism controlling the passage of urine. No other sphincter in the body has to be completely watertight for a normal social life in the community. The anal sphincter does not have to cope under normal conditions with a fluid content and a slight lack of control of the cardiac sphincter is in many parts of the world accepted as a compliment to the cook!

This intrinsic obstruction at the neck of the bladder can occur at all ages and in both sexes. We have seen it in the premature baby which leads us to believe in its true congenital origin and we suggest that a familial incidence may be sometimes found.

### HISTORY

In the past the condition has been recognized and described by many observers under many different names, but it was not appreciated that it was the same condition appearing at different times in life. Morgagni in his *Seats and causes of Diseases* published in 1760 must be given the credit of the first description when he writes about induration of the prostate gland. This, he observed, gave rise to urinary obstruction which in turn caused either chronic retention of urine or hypertrophy of the bladder wall with reduction in its capacity. Reading carefully the word-for-word translation of his work left me with some doubt as to whether his induration might not in some cases have had a malignant basis.

The first lucid understanding of this condition appeared in 1834 when Guthrie—one-time President of the Royal College of Surgeons of England—published his book entitled *The Anatomy and Diseases of the Neck of the Bladder* in which he devotes two entire chapters to chronic thickening of the neck. It is quite remarkable how little our knowledge has advanced since this first comprehensive study. In fact, his symptoms and signs were complete and his diagnosis in the more advanced cases was evidently accurate despite the lack of the cystoscope. He even noticed the association with pouches in the bladder wall and the likely risk of urinary infection. His treatment, however, was not as rational as his clinical observations and he wanders into the mysterious realms of potions, clysters, cupping, blistering and—most horrifying of all—the application of leeches to the perineum. He did

* Papers read to Bristol Medico-Chirurgical Society.

invent a barbarous form of internal urethrotomy knife which he used on four cases, but the reader is left guessing the results!

Mercier described the condition as an abnormal muscular projection of the posterior rim of the orifice of the bladder and this he called the valve of the bladder neck. Anything which irritated the bladder neck, such as chronic urethritis, was thought to produce spasm; this spasm resulted in hypertrophy of the musculature, which in the course of time was replaced by fibrous tissue.

In 1912 Hugh Young in America gave the first analysis of a large series of one hundred cases. All of these were treated by his direct-vision punch which, except for some mechanical and lighting improvements, was similar to the punch in use today.

The possible congenital origin of this condition was first suggested in 1928 by Herbst who regarded the fibrosis as a secondary change. Five years later Marion surveyed 77 cases but made few original observations. It remains a mystery to me why this condition of obstruction at the bladder neck should so frequently be referred to as Marion's disease, when others have made greater contributions towards its understanding. Marion *did* take full advantage of the French language which lends itself to vivid description, particularly of delicate subjects such as micturition.

The latest and most comprehensive survey was given by Badenoch of St. Bartholomew's Hospital in his Hunterian Lecture just after the last war. All his cases were associated with diverticulum of the bladder or bilateral hydronephrosis. In fact, he made the detection of one of these complications the criterion of diagnosis of bladder-neck obstruction.

The object of our papers is to show that a diagnosis can be made at an earlier stage in the condition before these complications have developed. I shall quote examples of the condition as it has been encountered in all age groups and in both sexes. It is possible in this way to build up a life history of the lesion.

### CLINICAL FEATURES

In the early stages this condition produces merely a disturbance of micturition, such as enuresis in the first three decades, or frequency and urgency in later life. With only slight obstruction the consequent hypertrophy of the wall results in a hypertonic bladder of small capacity. As the condition progresses urine is retained in ever-increasing quantities until ultimately a state of chronic retention with overflow incontinence is reached, or occasionally acute retention supervenes. This is regarded as stage two. Finally, the chronically distended bladder may cause back-pressure on the ureters and kidneys followed by hydronephrosis and renal failure. The upper urinary track may, however, be saved by the formation of a diverticulum of the bladder wall. In the past many of these unfortunate patients have been dubbed " neurotics " in the early stages. When the condition was more advanced they were diagnosed as having idiopathic atonic bladders or diverticula of unknown origin.

If the urine becomes infected at any stage in the progress of the disease the symptoms may be modified. In fact it is often an infective episode which first brings the patient to his doctor. Some patients have presented merely with recurrent attacks of pyelitis, cystitis or a somewhat indeterminate condition known as prostatitis.

During the past four years 314 cases have been examined and treated in the Urological Unit at Bristol.* In an earlier communication to the Royal Society of Medicine (Mitchell and Andrews, 1953), 73 cases were reported and most had been admitted under the care of Mr. Ashton Miller. The rest of the cases have been shared between us during the past two and a half years.†

* We are indebted to Mr. A. Wilfrid Adams for kindly allowing us to include a number of his patients whom he has treated in the past two years. We would also like to thank Mr. Adams for the advice and helpful criticism from his fertile imagination.

† I must thank Mr. Keith Chitty, senior registrar to the Department of Urology, who has given us invaluable help with this work in the past two years.

No case was included in this series if any of the following features were present:

1. Any significant prostatic enlargement palpable on rectal examination.
2. Any evidence of malignancy either clinically or histologically.
3. Any neurological signs which might account for a disturbance of bladder function.

After excluding all such cases the remaining 314 cases were accepted as true bladder-neck obstruction. There are in this series ten patients in whom the bladder-neck obstruction followed some months after an open operation for prostatectomy. I would here emphasize that rectal examination in all cases revealed a prostate which was quite normal both in size and consistency.

TABLE I

TEN-YEAR AGE GROUPS

| | | |
|---|---|---|
| Infancy | 9 | patients |
| 5–15 years | 55 | ,, |
| 16–25 years | 18 | ,, |
| 26–35 years | 27 | ,, |
| 36–45 years | 32 | ,, |
| 46–55 years | 58 | ,, |
| 56–65 years | 52 | ,, |
| 66–75 years | 48 | ,, |
| 76 years and over | 15 | ,, |
| Total | 314 | patients |

Table I shows the number of patients in each ten-year age group. It will be noticed that the incidence steadily increases up to the peak in the 46–55 decade; the figure is still maintained in the next decade but this is due partly to the cases of post-prostatectomy obstruction. It will be seen that the highest age incidence is therefore ten years prior to the age of onset of prostatic hypertrophy.

In the neo-natal period and in infancy the condition is seldom recognized unless retention occurs and the child is found to have a distended abdomen caused by a full bladder. One male infant was stated by the parents to go twenty-four hours without passing water, but what brought them to the doctor was the apparent straining and the crying just before micturition.

After the age of five the condition may be found as a result of investigations for recurrent attacks of urinary infection or for enuresis. A percentage of enuretics are found to have a true organic basis, of which bladder-neck hypertrophy is one. The usual history obtained in these cases is that the child was dry by night as well as by day from the age of three or four years, but after two or three dry years the child again relapsed into enuresis.

The child with slight obstruction proceeds to adult life with only slight disability. After puberty enuresis may disappear but frequency and urgency remain throughout life. These patients will say that they have always had a " small bladder " and they have never been able to last out the full programme at the cinema, even though they only see the programme through once. They may complain of difficulty and a poor stream with occasional attacks of pain on micturition. Hesitancy is often volunteered; the patient will tell you that he has to stand for several minutes before he can start in a public urinal and even when he has started two or three others will have relieved themselves completely before he has finished. In fact it is the story of the old prostatic gentleman appearing in a much younger age group. Most of the patients in the first two decades of adult life gave histories of enuresis in childhood after the age of six.

In the later age groups frequency is still the most common symptom though a form of incontinence may re-appear. This may be a true incontinence severe enough to be very distressing to the patient, but in others it is an overflow incontinence due to chronic retention. Acute retention brings some patients into hospital as emergencies, while occasionally investigation for haematuria reveals a large infected diverticulum. A symptom which is difficult to explain is the loin pain without frank infection or demonstrable dilation of the kidney. This is not uncommon and usually disappears when the bladder-neck obstruction is relieved.

The patient may reach the prostatic age group before his symptoms really inconvenience him. In fact, a moderate hypertrophy of the prostate may be sufficient to bring on symptoms in a patient who has only a mild degree of intrinsic bladder-neck obstruction; neither the small adenoma nor the milder bladder-neck obstruction alone would have taken the patient to his doctor. I would here point out that, though adenoma of the prostate and congenital bladder-neck obstruction are separate entities, they can occur simultaneously in the same patient. There are, however, still some cases of pure bladder-neck obstruction which first present at the prostatic age, having been slowly progressive throughout life. At this age and with no clinical or cystoscopic evidence of prostatic enlargement, this condition has been given the term " prostatisme sans prostate ".

In the female the symptoms are remarkably similar. In fact it is quite surprising sometimes to see how closely bladder-neck obstruction in the female resembles prostatism in the male. Infection is even more liable to occur than in the male and is usually of a more intractable nature.

### ENDOSCOPIC FINDINGS

To investigate these cases cystoscopically it is essential to use an irrigating cysto-urethroscope. The salient feature of this instrument is the short focal distance whereby objects as near as 3 mm. can still be in focus. By this means both the posterior urethra and bladder can be examined with the same instrument. Irrigation is necessary both to distend the posterior urethra where possible and to clear the field of vision. On introducing the instrument, difficulty may be encountered as the beak reaches the bladder neck and often the shaft of the instrument has to be dipped considerably before it will pass on into the bladder. The bladder is inspected for stone, diverticulum or neoplasm. The prostatic urethra is then viewed for evidence of early prostatic hypertrophy. Finally, the bladder neck itself is examined.

The presence of trabeculation is the cardinal sign in cytoscopic examination, and we believe that if the trabeculation persists on full distension of the bladder and is spread over the entire bladder wall, then it must be regarded as signifying some definite obstruction. At the bladder neck some degree of thickening may be seen but the localization of this thickening appears to vary. In some it extends from four to eight o'clock posteriorly, forming the classical median bar (Fig. 1). In others, particularly in children, it may be localized laterally at three and nine o'clock forming a pillar on each side of the bladder neck (Fig. 2). In the majority of cases a collar of thickening exists around the entire circumference of the internal urinary meatus (Fig. 3). As the cystoscope is withdrawn into the prostatic urethra the trigone appears to rise sharply up to the neck which then drops away equally sharply into the posterior urethra revealing a pouch below the bladder neck. This sometimes even undermines the bladder neck. If the trigone is also hypertrophied, these appearances will be modified.

The other helpful diagnostic sign is obtained with a finger *per rectum* at the time of the cystoscopy. The amount of tissue felt between the examining finger and the cystoscope being a guide to the degree of hypertrophy; only very occasionally can this bar be felt without an instrument present in the urethra. Figure 4 illustrates diagrammatically the thickening palpable against the cystoscope compared with the normal above.

FIG. 1. *Median bar.*

FIG. 2. *Lateral pillars.*

FIG. 3. *Collar thickening.*

*The appearances of the internal urinary meatus when viewed from the posterior urethra at the level of the verumontanum.*

FIG. 4. *The amount of tissue felt between the examining finger* per rectum *and the cystoscope at the bladder neck.*

[Figures published with permission of the Editor of the *Proceedings of the Royal Society of Medicine.*]

### TREATMENT

During the nineteenth century various instruments were designed to cut the bladder neck blindly, the only control being a finger *per rectum.* As already mentioned, Guthrie used an internal urethrotomy knife as early as 1830. Mercier improved on this with a punch designed on the lines of a lithotrite. Towards the end of the century Chetwood in America was resecting with a galvano-cautery knife.

In the early 1900's, Young was attempting to relieve bladder-neck obstruction by a perineal approach, but found this unsatisfactory particularly in young men in whom the whole prostate had to be sacrificed. He abandoned the perineal route in favour of the suprapubic transvesical approach. Finally, he designed his punch and was the first to resect the median bar transurethrally under direct vision.

His first operation was performed on an out-patient under local cocaine anaesthesia in 1909. By 1913 he reported the results of one hundred cases of median-bar obstruction which had been operated by transurethral approach.

In 1931 McCarthy brought out his modification of Stern's first loop-cutting machine and produced the first successful resectoscope. In the last twenty years various minor mechanical improvements have been made, but the principle of the machine is essentially the same.

If the symptoms are purely obstructive then the relief is immediate and dramatic, but if frequency is the chief complaint, then little improvement can be excepted within the first few weeks. The frequency is probably due to hypertonicity of the bladder with its reduced capacity. This bladder has a thickened wall and will take several weeks after operation to return to its normal shape and size. Patients should, therefore, be warned that relief of their frequency will not be immediate and the maximum benefit may not be noticed for three to six months.

The indications for surgical treatment were either the presence of complications such as hydronephrosis, diverticulum or chronic retention of urine, or the distressing nature of the symptoms in the earlier stages of the disease. Transurethral resection, using a valve machine for the cutting current, was the method of choice in most cases. The valve diathermy produces a current of much higher frequency than the spark-gap and this cuts the tissues like cheese with a minimum of charring. The extent of the resection necessary was judged from the appearance of the bladder neck and, in view of the narrow margin of error, a finger *per rectum* is essential in the final stage of resection. The transvesical approach is used in the event of additional intravesical pathology such as stones or diverticula. However, we are convinced that a more accurate clearance is obtained with the resectoscope than by open operation.

Every operation has its hazards, even when the patient has survived the anaesthetic, and transurethral resection is no exception. An error of judgment in the site and depth of a cut is liable to happen occasionally in the most experienced hands, but provided a perforation into the peri-vesical tissues is recognized and the appropriate action taken immediately, little harm can come. An adequate resection should remove sufficient tissue to destroy temporarily the action of the internal sphincter, just as happens after an open prostatectomy. This will result in sterility as the semen will pass into the bladder and not down the urethra with ejaculation. Usually this is only a temporary feature, but just occasionally permanent sterility may result and this is of some importance, particularly in the younger age groups.

It is not an operation to be undertaken lightly and should always be regarded as equal in severity to an open prostatectomy. Patients are always kept in hospital for twelve days to cover the period of secondary haemorrhage. Sometimes a minor flare-up of urethritis and prostatitis may occur even a week to ten days after returning home.

Cases of mild obstruction at the bladder neck may respond simply to dilation of the urethra though this usually gives only a temporary relief. It may, however, be sufficient to interrupt the vicious cycle of obstruction, mild chronic infection and further obstruction from oedema. I venture to suggest that this may explain the occasional curative value of a simple cystoscopy.

### ILLUSTRATIVE CASES

The following examples are shown to illustrate the points I have made and to show the condition in each decade of life:

1. **B.E.E.** A boy aged 7, who is a patient of Dr. Cameron of Bedminster, Bristol, was admitted to hospital in acute retention having had three previous attacks. He also had a history of enuresis every night since birth and investigations showed that he had a typical bladder-neck obstruction due to thickened latteral pillars (Fig. 2).

Transurethral resection was performed, since when he had had no enuresis.
Review one year after operation confirmed no further attacks of acute retention.

2. **K.F.R.** A lad of 17, referred by Dr. Chapman of Redland, Bristol, had had enuresis every night since birth and there was a family history of similar trouble in the father and one male cousin. He was an intelligent boy with a good scholastic record.

Investigation again showed thickened lateral pillars (Fig. 2), which were resected by the transurethral route.

Immediately after operation there was some improvement, in that he was dry provided he emptied his bladder at 4 o'clock every morning.

Eighteen months later a letter received from his mother stated that he had been completely dry for the past year.

3. **D.C.B.** A patient of Dr. Sinclair of Filton, Bristol, was referred by Mr. McPherson of Southmead Hospital.

He was a young man of 22 who presented with acute retention, having had scalding pain on micturition and a diminished stream for the past four years.

Investigation showed a grossly trabeculated bladder with a thick and raised median bar (Fig. 1).

Transurethral resection relieved the retention, and when seen eight months after operation he had a good free stream and no pain on micturition. At that stage he had not yet recovered normal ejaculation.

4. **C.C.R.** Aged 37. A patient of Dr. Kevin Burke of Patchway, Bristol, presented with a history of enuresis all his life and frequency amounting to half-hourly by day and six times at night.

Cystoscopy showed a typical bladder-neck obstruction due to thickened lateral pillars (Fig. 2).—

A transurethral resection was performed which gave immediate relief of his enuresis.

Three months after operation his frequency amounted to only four-hourly by day and once or twice at night, but ejaculation was still absent.

I have recently received a letter from him telling me that he is now symptom-free and ejaculation is once again normal.

5. **K.W.H.** Aged 49, was referred by Dr. Woolley of Wells, Som. He presented with a history of frequency one-hourly by day and two to three times at night for the past ten years. He also had slight but aggravating right-loin pain.

Investigation showed a normal upper urinary tract, but cystoscopy revealed a trabeculated bladder with a typical median-bar obstruction (Fig. 1).

A transurethral resection was performed but this gave no improvement for the first three months.

At the end of nine months, however, his frequency was only two to three-hourly by day and nil at night. He also volunteered the information that his stream is now much freer than ever before.

This case illustrates the delay in recovery if the presenting symptom is simple frequency.

6. **H.J.S.** Aged 65, was referred by Dr. Henderson of Thornbury, Glos. This patient, in the prostatic age group, presented with frequency, difficulty, hesitancy and pain on micturition. He had also had recurrent attacks of epididymitis.

On investigation his urine was heavily infected, he had a small round thick-walled bladder and a thickened collar at the bladder neck (Fig. 3).

Transurethral resection gave immediate relief of the difficulty but he had persistent frequency and pain on micturition for four months after operation.

When seen fifteen months after his operation he was symptom-free and the urine was clear.

7. **J.J.M.** Aged 77, was referred by Dr. Mahood of Bishopston, Bristol. This old gentleman presented with a history of hesitancy, poor stream and pain on micturition for twenty years. His bladder was distended up to the umbilicus, soft and painless and the urine was clear.

Cystoscopy revealed a typical median bar with no trace of prostatic hypertrophy.

Transurethral resection was complicated by a post-operative epididymitis, but one year later he was very well, passing water freely with a good stream and frequency amounting to two- to three-hourly by day and nil at night.

This is a typical example of "prostatisme sans prostate".

Many normal people have an occasional disturbance of micturition often precipitated by some emotional stress. Some of us may have experienced hesitancy in starting to pass water during the interval at Twickenham when there is a crowd waiting behind, and you may ask me whether this constitutes bladder-neck obstruction. Well, maybe it is in a mild degree, but obviously the limited inconvenience does not justify a resection of the bladder neck!

Clearly it is not easy to assess where the normal ends and pathology begins in many branches of medicine, but I have tried, this evening, to convince you that there *is* a stage in this condition before hydronephrosis occurs or diverticula of the bladder are produced, in fact before even a residual urine is obvious. In this early stage the symptoms can be very distressing and for these patients much can be done. By far the majority of them will be every bit as grateful as the old gentleman who has had an uneventful prostatectomy.

### PART II

### BY ASHTON MILLER

Mr. Mitchell has given you a paper most of which is fact and very little fancy. It is my part to deal with the condition of bladder-neck obstruction as it is found in children and in women—not so much will therefore be fact and rather more fancy, as you might expect. One reason for this difference is, of course, that methods of accurate cystoscopic and radiographic examination in young children have been available for only about ten years since men like Meredith Campbell in the United States led the way a few years before. Another reason is, possibly, that intrinsic diseases of the female urethra have long been just a little beneath the dignity of the surgeon, the urologist and possibly the gynaecologist also.

We have been able to carry out detailed examination of the urethra in children and women during the last ten years and it leaves no doubt at all in our minds that this condition of bladder-neck obstruction exists in the child of both sexes and in the adult woman as well as in the adult male.

### BLADDER-NECK OBSTRUCTION IN CHILDREN

Let us consider the children first: the earliest age at which we have been able to confirm the diagnosis of bladder-neck obstruction has been in a baby that was born three weeks prematurely with a distended bladder. Another case was recognized ten days after a full-term delivery:

**Baby O.** Born at Southmead Hospital, an apparently normal male child which lost weight continuously. The abdomen was noticed to be distended and on the eleventh day this distention was found to be due to a large bladder; there was a bilateral hydronephrosis and hydroureter and the blood urea was then 55 mg. per cent. Urine dribbled away *per urethram* constantly.

Operation was performed on the twelfth day. The bladder was grossly hypertrophied and the internal meatus narrow. No urethral valves could be demonstrated by passage of a sound down the urethra from the bladder. The posterior half of the thick bladder neck was excised. Convalescence was uneventful and subsequently he voided urine normally without residual urine in the bladder.

At the present date his micturition is normal but there is still a moderate bilateral hydronephrosis with a slightly raised blood urea level.

**Baby L.** Born three weeks prematurely and found to have a distended bladder and to pass only a few drops of urine. Immediate operation was performed and a similar portion of bladder neck resected.

Progress has been satisfactory after a period when recurrent urinary infections were a nuisance.

It is pleasant to thank Dr. Beryl Corner and other paediatricians for referring these cases to us.

These cases make it reasonably certain that the condition occurs in the foetus and is therefore congenital. We can also draw the conclusion that, because hypertrophy of the bladder due to urethral obstruction can occur in the foetus, the normal infant's bladder fills and empties *in utero* and micturition into the amniotic fluid occurs. It is obvious that the maternal kidneys do most of the work of electrolyte and urea excretion but it is possible that the foetal kidney could be concerned with fluid balance in the baby.

Other neo-natal cases have been seen and treated during the past few years with a similar history to those already given.

These infants have varying degrees of urinary obstruction with secondary renal failure. It is unusual for anybody to notice anything wrong with the micturition of a baby—even retention with overflow sometimes goes unrecognized because the baby is " always wet " and most people think that babies pass urine so often that they can be normally always wet! A distended bladder is sometimes felt by a doctor who is looking for it but rarely noticed by a parent or even a nurse. Usually it is the onset of renal failure which allows the diagnosis to be made and this is shown by the failure to gain weight, a disinterest in feeding—whether breast or bottle—and occasional vomiting after feeds. This picture can continue for weeks or even months and then suddenly the circle becomes vicious, vomiting and diarrhoea usher in dehydration which effectively stops completely the actions of the kidneys with rapid progress in a few hours to coma and death—the typical sudden death which occurs in chronic renal failure. The clue to these cases is the distended bladder and, of course, successful treatment of the urinary obstruction depends upon early diagnosis in relation to the course of the disease. It is true that a distended bladder may be easily felt in a normal baby on occasions because at that age it is an abdominal organ when full, but when micturition occurs it empties completely.

Another surprising way in which this condition can present in the child is by acute retention of urine; this is painful and just like that in an adult but it is often relieved spontaneously in a hot bath which allows micturition to start and the bladder is completely emptied—just as if the trigger is too stiff to pull and needs oiling. Passage of a catheter into the anterior urethra will sometimes start micturition in these patients; the hypertrophied sphincter is apparently in spasm. Here is a history of such a case:

**Baby H., aged 18 months.** This child had been admitted to hospital elsewhere on three occasions with acute retention of urine. On each occasion urethral instruments were passed and the retention was relieved; at the last admission the surgeon, Mr. Edrich Wilson, felt a bridge of tissue on rectal palpation against the cystoscope and referred him to the Children's Hospital. Investigation has confirmed the bladder-neck obstruction and has shown in addition that there is ureteric stenosis on both sides. Suitable operative treatment is in progress.

For every dramatic case such as I have described there are undoubtedly quite a large number of mild cases which escape notice until the age of 3 to 5 years. At this time they are commonly enuretic. Typically they have a little frequency in the day-time with some urgency—Daddy must stop the car immediately on demand, there is no question of choosing a nice wood in the next mile or two. It is this which gets them labelled " incontinent "; in the lay sense of the word they are, of course, but in the medical sense they simply must micturate when they have the desire and are not truly incontinent in the sense that anything that runs into the bladder immediately runs out because there is no adequate sphincter mechanism. A familiar complaint is that the teacher at school will not let the child go out to pass urine when he wants to. Many schoolteachers are extraordinarily helpful and allow these children to go out without asking each time; which is surely the best and kindest way. This urgency of micturition—a hair-trigger this time—occurs whether they are awake or asleep.

**Case of S.P., aged 3 years,** referred by Dr. A. L. Pirrie. Chief complaint was dribbling micturition. He had always had frequency and difficulty and had never passed a proper stream of urine. He was enuretic every night.

Examination showed a healthy-looking child with a bladder distended to the umbilicus. He voided urine with a dribble by straining. The urine contained many pus cells and organisms. The blood urea was normal. Intravenous pyelography revealed a functionless right kidney and a hydronephrosis on the left side.

Cystoscopy showed a thick bladder neck and a trabeculated bladder. The right ureteric orifice was wide open but the left one was normal. There was a diverticulum on the left side of the bladder. Suprapubic cystostomy was first performed and these findings confirmed. Three months later the functionless right kidney and ureter were removed and excision of the bladder neck followed shortly afterwards. Progress to normal micturition was rapid but recurrent attacks of cystitis occurred until the diverticulum was removed a year later. Since then he has been dry both during the day and at night, being last seen five weeks ago. A recent pyelogram shows that the hydronephrosis is smaller and that the ureter has returned to normal.

You will ask me how many enuretic children have bladder-neck obstruction and I must answer simply that I cannot give you a figure or a percentage yet and can only say that a significant number have it. In 1951, Dr. Mary Boyd, Registrar at the Bristol Children's Hospital, investigated many enuretic children and combined for the first time the reports of the paediatrician, the psychiatrist, the urologist, the radiologist, even the results of the electro-encephalograms. The results have not all come to light yet but I can tell you what we found in the urinary systems.

All told 84 boys and 45 girls were investigated between the ages of 5 and 13 years inclusive. These were patients referred to the Children's Hospital and the method of selection out of the total number of enuretics in the child population was not accurately known. For example, no conclusion can be drawn from the fact that twice as many boys as girls were examined—this may be accidental; also we may well have encountered the worst enuretics.

Between the ages of 5 and 9 years one-quarter of those examined had an abnormal bladder, viz., 28 out of 90; between 10 and 13 years a higher proportion were abnormal, viz., 14 out of 34, or about one-third. The abnormality consisted of a definite hypertrophy of the bladder wall seen through the cystoscope combined with a thickening of the internal sphincter at the bladder neck palpable by the finger *per rectum* against the cystoscope shaft—a method which Mr. Mitchell invented and which reduces the hazard of personal error in observation. We believe that these children have bladder-neck obstruction which results in a bladder muscle hypertrophy, producing a hypertonic bladder which requires more central inhibition both awake and asleep than exists normally at this age. In only one case have we found a possible urethral valve as was recently described by Innes Williams (1954).

We would stress, though, that these proportions are not accurate, we can only say that there is no doubt at all in our minds that it is a mistake to treat a case of persistent enuresis in an otherwise normal child over the age of five without urological examination to exclude organic urinary obstruction, especially when such clues as, for example, frequency and urgency by day and inability occasionally to sleep through a whole night and be dry, exist to lead us to the correct diagnosis. It is not so much that these patients should be treated surgically—far from it—it is not fair to the psychiatrists to give them a child with an organic lesion to work on.

It is an interesting fact that bladder-neck obstruction can very occasionally exist in a child without enuresis—the child wakes up at night and gets out of bed to pass water. We do not know why this happens. Sometimes, not really uncommonly, attacks of urinary infection are the reason why the child comes to the doctor; we have found this is mostly true of girls, and this observation also applies when the condition is met in the adult woman. A recurrence of enuresis after the child has been quite dry at night for several months or years indicates in every case in our experience a true organic bladder-neck obstruction.

We have unconsciously, perhaps, divided the cases into slight, moderate and severe, slight being common and usually needing no surgical treatment, moderate is not so common and has not usually been treated by surgery, severe is rare but always needs surgical treatment. The slight cases appear always to lose their symptoms and stop enuresis at puberty if not shortly before, though it is possible, of course, that they may have trouble later in life. The moderate cases are not so fortunate for enuresis often persists for some time into adult life and other vague symptoms related to micturition and the genitalia may cause the diagnostic refuge of neurosis to be sought. We have always cystoscoped the child first and then waited a few months to see if there is any improvement; in bladder-neck obstruction improvement seldom occurs and, if it does, relapse is common within a short time. If the child is enuretic and we find the bladder and urethra normal, then it is sometimes times a surprising and gratifying result of instrumentation that enuresis ceases from that day on. You can speculate as much as you like as to why this occurs; possibly Mr. Mitchell's explanation may be the right one—we do not know. All I can tell you is that it has been known as an empirical treatment of enuresis for several generations. The uncomfortable micturition which follows cystoscopy for about twenty-four hours may certainly be a powerful suggestive factor.

### SURGICAL TREATMENT

Let me tell you something of the operation which is done. In the neo-natal cases all operating has to be done through the open bladder and a pair of conjunctival scissors is used to remove a large part of the internal sphincter at the outlet of the bladder, only the periphery of the muscular ring being left behind. The bladder is then closed leaving a minute catheter in the urethra. None of this surgery in the neo-natal period would be possible without the exceedingly skilled co-operation of the anaesthetist so I would like to thank Dr. Torry Baxter for his remarkably efficient and successful help with all these babies that we have operated on. The nursing is also a highly skilled job to which I would like you to give a thought. In both the male and female child over the age of 2 years it is possible to perform the same operation transurethrally with an instrument which is a small version of the resectoscope we use in adults. This was specially designed for use at the Children's Hospital. The female urethra can be stretched a good deal without harm—it is recorded in an old French urological work that a finger could be inserted into the dilated adult female urethra in order to palpate a calculus in the lower end of the ureter against a finger of the other hand in the vagina. Today the radiologist has given us a more refined and less traumatic method, so the recollection does no more than remind us that dilatation is permissible in the female. In the male this is emphatically not so. Luckily there is a way round the difficulty because the bulbous urethra in the perineum is easily wide enough in the child and adult to take quite large instruments; so we introduce them direct through the perineum, do the operation and then allow the hole to close up again, which it usually does in about 48 hours with no leakage afterwards and no stricture formation. This is an arrangement undoubtedly thought of by nature for the special benefit of urologists, otherwise everyone of us would be permanently followed by a stream of old patients with urethral strictures shaking their fists and saying—" Look what you did to me, doctor! "

The margin of error in this operation in a child is small but vision is good—it has to be—and a finger *per rectum* certainly makes it safer.

The results have certainly justified the operation; we have performed it 33 times in children without untoward result. One child is certainly no better but all the others have shown improvement and in some cases the result has had a fine dramatic quality in that enuresis has stopped from the day of the operation and not recurred. In some cases we have erred on the side of caution and removed too little and had to do the operation a second time. It is satisfactory in a way to be able to prove that this condition exists by removing the obstruction and producing a cure.

### BLADDER-NECK OBSTRUCTION IN WOMEN

In the adult woman we see this condition in the twenties, thirties and forties, but it does not seem to be such a common cause of symptoms as it is in men in these decades. The usual picture is of recurrent attacks of urinary infection, the accent being on the lower urinary track rather than on a pyelonephritis, in other words they have frequency, nocturia and pain with micturition as their chief symptoms, rather than pyrexia, rigors and pain in the back. I do not mean to suggest that pyelonephritis never occurs as a complication for this would not be true; I am sure that recurrent attacks of urinary infection in the female are worth investigation and efficient treatment, not only because of the disability caused but also because of their long-term results, chronic pyelonephritis with renal failure with or without hypertension and the unpleasant complications of pregnancy which can occur—and often be more lethal to the child than the mother.

We have to admit though that we can never diagnose a bladder-neck obstruction in an adult female before cystoscopy as it is possible to do sometimes in the male. Adult enuresis may occur but is very uncommon indeed in women, the vast majority of cases may have been enuretic as a young girl and then have apparently normal micturition until a sudden attack of urinary infection occurs and recurs after treatment. Investigation by clinical examination and pyelography shows nothing abnormal and it is only the cystoscopy that reveals the diagnosis. But we probably cystoscope twenty normal patients who have this story for every one who has a bladder-neck obstruction—it appears that identical symptoms can exist in the female without an underlying organic abnormality. Also, it seems that recurrent attacks of urethritis in the female can produce an inflammatory fibrosis with stricture formation, resulting in urinary obstruction and the same symptoms. Cysto-urethroscopy easily distinguishes the two conditions in their early stages but there is more than a little confusion because it seems that the two conditions can exist together in the more elderly patient.

We have investigated and treated twenty-one examples of the condition in the adult female, performing a transurethral resection in all cases. The results are not as good as in the male, possibly because the field is so often clouded by other things such as prolapse and cervicitis which themselves result in urinary dysfunction. There is no doubt that in the early uncomplicated case the results are excellent.

Here is the case history of a typical example:

**Patient Miss G. aged 20.** She had been enuretic every night of her life. She micturated about every hour during the day and urgency was marked. Her brother had died of kidney trouble at the age of four years. There were no abnormal physical signs on examination. The urine was normal. Intravenous pyelography demonstrated normal kidneys with stasis in the lower ureters. Cystoscopy showed marked trabeculation of the bladder. Transurethral resection of the bladder neck was performed and she wet the bed only thrice in the first month after operation.

This case raises the fascinating question of the familial incidence of this condition. We have the distinct impression that it really is familial but, of course, have not had sufficient opportunity to verify this.

In contrast to this last case history let me give you a typical example of the infective type in a woman of 41:

Eight years ago she had an attack of "cystitis" following exposure to cold, with several recurrences in the next two years, treated successfully with sulphonamide. Cystoscopy and urethral dilatation were performed without much relief. She saw two London surgeons who performed dilatation, and then she saw another local surgeon who performed diathermy coagulation of the bladder neck with some relief for about a month. She then had to micturate twice or more at night. A course of N.A.B. was given without benefit.

Cystoscopy showed normal bladder mucosa with thickened urethra when palpated against the cystoscope.

Transurethral resection of the bladder neck was performed. Histology showed chronic inflammatory fibrosis. Four months later, after a stormy convalescence, cystoscopy showed an almost normal urethra. Shortly afterwards her symptoms disappeared, and micturition to normal.

Not all patients are so typical as these two women who serve just as illustrations. In cases such as these we feel ourselves on sure ground, but in many, I regret to say, there is no such feeling and we are reminded once again that we have a lot to learn.

### REFERENCES

Badenoch, A. W. (1949). *Ann. R.C.S.*, **4**, 295.
Guthrie, G. J. (1834). *The Anatomy and Diseases of the Neck of the Bladder*, Burgess and Hill, London.
Herbst, R. H. (1928). *J. Amer. med. Ass.*, **91**, 1614.
Marion, G. (1927). *Journal d'Urologie*, **23**, 97.
Mercier, L. A. (1856). *Recherches sur le traitment des maladies des organes urinaires*, Paris.
Mitchell, J. P. and Andrews, G. S. (1953). *Proc. Roy. Soc. Med.*, **46**, 549.
Morgani, J. B. (1760). *The Seats and Causes of Disease.*
Williams, I. (1954). *Brit. M. J.*, **1**, 623.
Young, H. H. (1913). *J.A.M.A.*, **60**, 253.

## Figures and Tables

**Fig. 1. Fig. 2. Fig. 3. f1:**
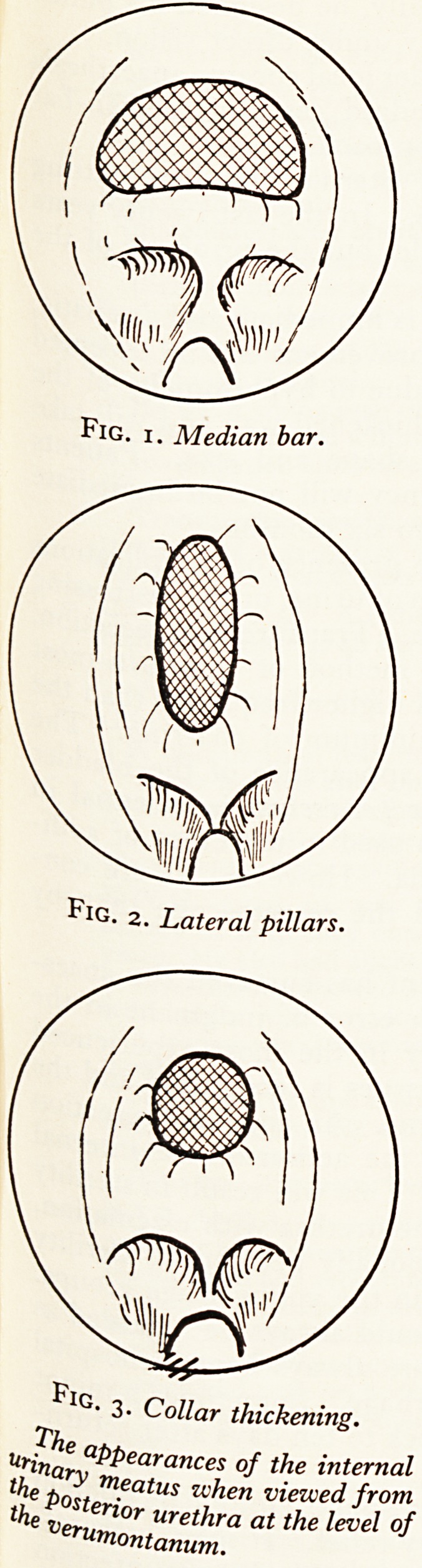


**Fig. 4. f2:**